# β-Xylosidase and β-mannosidase in combination improved growth performance and altered microbial profiles in weanling pigs fed a corn-soybean meal-based diet

**DOI:** 10.5713/ajas.18.0873

**Published:** 2019-02-14

**Authors:** Shaoshuai Liu, Chang Ma, Ling Liu, Dong Ning, Yajing Liu, Bing Dong

**Affiliations:** 1 State Key Laboratory of Animal Nutrition, College of Animal Science and Technology, China Agricultural University, Beijing 100193, China; 2 Asiapac Limited Company, Dongguan, Guangdong 523808, China

**Keywords:** β-Xylosidase, β-Mannosidase, Piglet, Growth Performance, Soybean Meal, Corn

## Abstract

**Objective:**

In this study, two glycosidases (XMosidases), β-xylosidase and β-mannosidase, were investigated on their *in vitro* hydrolysis activities of feed and on the improvement of growth performance *in vivo* in weanling pigs.

**Methods:**

Enzyme activities of XMosidases *in vitro* were evaluated in test tubes and simulation of gastric and small intestinal digestion, respectively, in the presence of NSPase. *In vivo* study was performed in 108 weaned piglets in a 28-d treatment. Pigs were allotted to one of three dietary treatments with six replicate pens in each treatment. The three treatment groups were as follows: i) Control (basal diet); ii) CE (basal diets+CE); iii) CE-Xmosidases (basal diets+ CE+β-xylosidase at 800 U/kg and β-mannosidase at 40 U/kg). CE was complex enzymes (amylase, protease, xylanase, and mannanase).

**Results:**

*In vitro* XMosidases displayed significant activities on hydrolysis of corn and soybean meal in the presence of non-starch polysaccharide degrading enzymes (xylanase and β-mannanase). *In vitro* simulation of gastric and small intestinal digestion by XMosidases showed XMosidases achieved 67.89%±0.22% of dry matter digestibility and 63.12%±0.21% of energy digestibility at 40°C for 5 hrs. In weanling pigs, additional XMosidases to CE in feed improved average daily gain, feed conversion rate (p<0.05), and apparent total tract digestibility of crude protein (p = 0.01) and dry matter (p = 0.02). XMosidases also altered the gut bacterial diversity and composition by increasing the proportion of beneficial bacteria.

**Conclusion:**

Addition of a complex enzyme supplementation (contained xylanase, β-mannanase, protease and amylase), XMosidases (β-xylosidase and β-mannosidase) can further improve the growth performance and nutrient digestion of young pigs.

## INTRODUCTION

Corn and soybean meal (SBM) based feed is commonly utilized in pork production, yet plant sourced feedstuffs contain numerous components such as cell walls that are resistant to digestion. The cell wall is a highly integrated structure composed of various polysaccharides, proteins and cross-linked compounds. Mammals either lack, or produce insufficient amounts, of endogenous enzymes to digest these components. Thus, to fully utilize plant origin feedstuffs, a range of enzymes have been produced that are included in the feed, such as proteases, amylases, and carbohydrates, to aid in digestion of nutrients [1–3].

Non-starch polysaccharides (NSPs) contained in corn based diets are especially resistant to the action of NSP endo-acting xylanases and glucanases [4]. The most prevalent NSP in corn, as in many cereal grains, is arabinoxylan (AX). Corn AX has a β-1,4-linked xylose backbone with arabinose units attached. Additional residues include glucuronic acid, xylose, and galactose, are also attached to the structure. Specifically, hydroxycinnamic acid residues and acetyl groups attached to both the branches (arabinose) and the backbone (xylose), render corn AX much more resistant to digestion by xylanase [5,6]. Thorough digestion of corn NSPs requires a spectrum of enzymes. Xylanase (EC 3.2.1.8) can degrade xylan by randomly hydrolyzing the β-1,4-glycosidic bonds between xylose residues producing different lengths of xylo-oligosaccharides. Xylan 1,4-β-xylosidase (EC 3.2.1.37) can catalyze the hydrolysis of (1,4)-β-D-xylans by removing successive D-xylose residues from the non-reducing end of the xylose oligomers arising from endoxylanase activity. β-Xylosidase has been reported to be rate-limiting in xylan hydrolysis [7] and is important for complete hydrolysis of xylan.

The SBM is a soybean oil extraction by-product that has become the major protein source in livestock feeds. SBM contains significant amount of polysaccharides (15% to 22%), such as acidic polysaccharides, arabinogalactans, and cellulose [8]. β-Mannans is a heat insensitive antinutritional component of SBM and makes up approximately 1% to 2% of the dry weight of SBM. It remains stable during the heat and drying procedures of feed processing [9]. β-Mannans are linear polysaccharides composed of repeating units of β-1,4-mannose or β-1,4-linked mannose and glucose residues [10], with alpha-1,6-linked galactose in some forms [11]. Hydrolysis of mannans requires a unique set of enzymes. β-Mannanase (EC 3.2.1.78) is pivotal to initiate the degradation of mannans by randomly cleaving the backbone of mannans. β-Mannosidases (EC 3.2.1.25) perform the next step of hydrolysis by exo-cleaving β-1,4-linked mannosides to release mannose from mannan oligosaccharides or the non-reducing end of mannans [10,11]. Mannobiose and mannotriose have been reported as the main products of β-mannan digestion [12,13].

A balanced intestinal micro flora contributes to a healthy body condition [14]. In cereal grain-based diets, high NSP contents generate high digesta viscosity, which decreases nutrient digestion and absorption. This enhances microbial fermentation in the hind gut of the animal. Supplementation of enzymes can help aid digestion and nutrient absorption. Simultaneously, better digestion reduces the digesta viscosity and speeds the chyme emptying rate. The altered substrates and fermentation activity can thereby change the gut micro flora composition and microbial metabolite production [15, 16].

In this study, we evaluated the effect of additional two types of glycosidases (XMosidases: β-xylosidase and β-mannosidase) on the basis of multiple enzymes (xylanase, β-mannanase, protease, amylase) in corn and SBM based diets. The evaluation was conducted both *in vitro* and *in vivo*. *In vitro*, we examined the hydrolysis activities of XMosidases on SBM and corn. In the *in vivo* study, the effects of XMosidases on growth performance, gut microbial composition and volatile fatty acid (VFA) and lactic acid concentration in cecum and colon digesta were investigated. The hypothesis of this study was to test if the addition of XMosidases could increase the utilization of feed and thereby further improve growth performance.

## MATERIALS AND METHODS

### Enzymes and activity assay

Enzymatic hydrolysis of feed ingredients was assayed in reaction mixtures containing 15 mL of 0.1 M phosphate sodium citrate buffer (pH 6.0), 15 mL double distilled water, 1 mL penicillin, 0.5 mL chloramphenicol, 11 mL pancreatin, 1 g feed ingredients and 100 μL of appropriately diluted enzymes (5 U of xylanase, 5 U of β-mannanase, 5 U/10 U of β-xylosidase and 5 U/10 U of β-mannosidase). The pH of the reaction mixture was adjusted to 6.5, and incubated at 40°C for 5 hrs. The reducing sugar content was determined using dinitrosalicylic acid [17]. Non-starch polysaccharide enzymes (NSPase), xylanase (EC 3.2.1.8, Trichoderma viride) and β-mannanase (EC 3.2.1.78, *Bacillus circulans*) were purchased from Megazyme (Bray, County Wicklow, Ireland). XMosidases of β-xylosidase (EC 3.2.1.37, Aspergillus niger) and β-mannosidase (EC 3.2.1.25, Rhizomucor miehei) were purchased from Asiapac (Dongguan, China). Feed ingredients were a mixture of corn (70% w/w) and SBM (30% w/w). Feed was finely ground through a 0.42 mm size screen and pretreated at 90°C for 2 hrs, for inactivation of endogenous xylanase and its inhibitors, prior to the hydrolysis reaction. Each reaction was performed in triplicate.

### *In vitro* simulation of gastric and small intestinal digestion by XMosidases

Using the method reported by Chen [18], 2 g of feed ingredients were mixed with gastric enzyme (1,550 U/mL of pepsin; Sigma-Aldrich, St. Louis, MO, USA), appropriately diluted enzymes (NSPase: 5 U of xylanase, 5 U of β-mannanase, and XMosidases: 5 U/10 U of β-xylosidase, 5 U/10 U of β-mannosidase) and added to the dialysis tubes in the digestion chambers of Bionic Digestive System of Monogastric Animals (SDS-II, Shenhua Biotech LTD., Guangzhou, Guangdong, China). Tubes were incubated in a shaking bath (180 rpm at 39°C) for 4 hrs followed by three washes, each with 1,500 mL gastric buffer (40 min/wash). The digestion chambers contained 88.5 mmol/L of NaCl, 6.6 mmol/L of KCl, and 10 mmol/L of HCl (pH 2.0) to match the *in vivo* ionic concentration of gastric fluid of growing pigs. A similar method was performed for the small intestinal digestion. An enzyme solution containing 221.4 U/mL of amylase (Sigma-Aldrich, USA), 69.1 U/mL of trypsin (Amersco, Solon, OH, USA), 8.7 U/mL of chymotrypsin (Amersco, USA), NSPase, and XMosidases was injected into the tubes following gastric digestion described above. The small intestinal buffer in the digestion chambers contained 98.7 mmol/L of NaCl, 16.4 mmol/L of KCl, 170 mmol/L NaH2PO4, and 30 mmol/L Na2HPO4 (pH 6.44) to match the *in vivo* ionic concentration of small intestinal fluid from growing pigs [19]. The incubation time was 16 hrs at 39°C followed by six washes each with 1,500 mL small intestinal buffer (40 min/wash). The undigested residues in the dialysis tubes were dried overnight at 65°C and then incubated at 105°C for 5 h before analysis [20].

*In vitro* dry matter digestibility (IVDMD) was calculated by the formula:

$$\text{IVDMD }(\%)=({\text{M}}_{0}-{\text{M}}_{1})/{\text{M}}_{0}\times 100\%$$

*In vitro* digestive energy (IVDE) was calculated by the formulation:

$$\text{IVDE }(\%)=[({\text{M}}_{0}\times \text{ingredient gross energy }[\text{GE}])-({\text{M}}_{1}\times \text{residue GE})]/({\text{M}}_{0}\times \text{ingredient GE})\times 100\%$$

where M_0_ is the weight of feed ingredient before hydrolysis; M_1_ is the residue weight in dialysis tube.

### Animals, facilities, and experiment diets

The animal performance study was conducted at Swine Nutrition Research Centre of National Feed Engineering Technology Research Centre (Chengde, Hebei, China). All animal procedures used in these experiments were approved by the Institutional Animal Care and Use Committee of China Agricultural University (Beijing, China). One nursery barn was used in the study. The barn was a closed facility with mechanical ventilation equipped with 36 pens and 6 pigs (three barrows and three gilts) per pen resulting in 0.45 m^2^/pig (1.8 m×1.5 m divided by 6). The floor was one-half slatted concrete. Each pen was equipped with 1 nipple waterer and 1 feeder. A total of 108 crossbred (Duroc×Landrace×Yorkshire) pigs, with an average initial body weight (BW) of 7.87±1.56 kg, were blocked according to gender, ancestry and BW. Pigs were allotted to one of three dietary treatments with six replicate pens in each treatment. The three treatment groups were as follows: i) Control (basal diet); ii) CE (basal diets+complex enzymes); iii) CE-Xmosidases (CE+β-xylosidase at 800 U/kg and β-mannosidase at 40 U/kg). Enzymes were added after feed processing, because some processing steps decrease enzyme activity. The basal diet, comprised of corn, SBM, soy protein concentrate and dried whey, was formulated to meet or exceed NRC [21] requirements for weanling piglets (Table 1). Chromic oxide (3,000 mg/kg) was included in all diets as an inert marker. Complex enzymes (CE) are a commercial product comprised of xylanase (4,000 U/g), β-mannanase (500 U/g), protease (1,500 U/g), and amylase (1,500 U/g). The dose of CE supplementation was 1 g/kg feed. Pigs had *ad libitum* access to feed and water for the duration (28 days) of the experiment. The occurrence of diarrhea was observed by visual evaluation. Every day at 8:00 am and 4:00 pm, piglets were examined one by one for fecal contamination and swelling. The score of fecal consistency was that 0 = shaping or granular; 1 = soft stool shaping; 2 = dense, amorphous, fecal water without separation; and 3 = liquid, amorphous, and fecal water with separation. Fecal consistency score at grade 2 or 3 for 2 consecutive days was defined as diarrhea. Diarrhea rate (%) = the total number of days of diarrhea in piglets in single treatment group/(total number of piglets in single treatment group×28 d)×100%.

Feed disappearance was recorded daily by weighing out any residual feed from the previous day prior to adding new feed, and was defined as average daily feed intake (ADFI). Average daily gain (ADG) was calculated as total weight gain of pigs within a given pen divided by number of experimental days. Gain to feed ratio (G:F) was calculated by dividing ADG with ADFI. Feed samples for each treatment were collected from every batch of feed produced, pooled and mixed within treatment. Fresh fecal samples were taken from each pen on d 27 and 28 of the experiment and frozen for later analysis. Fecal samples were collected at least six times a day from the floor of each pen. The digestibility of various chemical constituents and apparent total tract digestibility (ATTD) were calculated as reported previously [22]. On d 28 of the experiment, one pig from each pen was selected to be slaughtered. Digesta of ileum, cecum and colon were collected aseptically and immediately immersed in liquid nitrogen and stored at −80°C for further analysis.

### Chemical analysis

Feed and fecal samples were analyzed according to the methods of the Association of Official Analytical Chemists [23,24]. The GE was measured via an adiabatic oxygen bomb calorimeter (Parr Instruments, Moline, IL, USA). Chromium concentrations of diets and fecal samples were determined after nitric acid-perchloric acid wet ash sample preparation using a Polarized Zeeman Atomic Absorption Spectrometer (Hitachi Z2000, Tokyo, Japan). All analyses were performed in duplicate and repeated when the results differed by more than 5%.

### Analysis of volatile fatty acids and lactic acid in digesta

Digesta samples were thawed at 4°C and thoroughly mixed immediately before testing. The concentrations of VFA were determined via ion chromatography (ICS 3000, Thermo, Wilmington, DE, USA). Briefly, about 1 g of thawed digesta sample was suspended in 8 mL of distilled water in a centrifuge tube and subjected to ultrasonic irradiation for 20 min, followed by centrifugation for 15 min at 10,000×g at 4°C. A sample (0.8 mL) of supernatant was mixed with 7.2 mL distilled water. The supernatants were diluted appropriately and analyzed by ion chromatography (Ionpac AS11, Waltham, MA, USA). The VFA concentrations were normalized to digesta weight as mg/g.

### Statistical analysis

Data were analyzed using one-way analysis of variance in accordance with the general linear model procedures of SAS 9.2 (SAS Institute Inc., Cary, NC, USA) utilizing a randomized complete block design by weight, including the terms for treatments and blocks with pen deemed as the experimental unit. Interactive matrix language procedure (IML) of SAS was adopted to generate the coefficients of unequally spaced contrasts. Significance level was set at p<0.05.

### Microbial diversity analysis

DNA extraction and polymerase chain reaction (PCR) amplification: digesta samples of six replicates from treated groups were blended in equimolar ratios based on concentration for PCR amplification. Total bacteria genomic DNA extraction was performed from ileum, cecal and colonic specimens by use of the E.Z.N.A. Stool DNA Kits (Omega Biotek, Norcross, GA, USA). The final DNA concentration and purification were determined by NanoDrop 2000 UV-vis spectrophotometer (Thermo Scientific, USA), and DNA quality was checked by 1% agarose gel electrophoresis. The V4-V5 region of the bacterial 16S ribosomal RNA gene was amplified by PCR (95°C for 2 min, followed by 25 cycles at 95°C for 30 s, 55°C for 30 s, and 72°C for 30 s and a final extension at 72°C for 5 min). The primers used were 338F 5′-barcode-ACTCCTACG GGAGGCAGCAG-3′ and 806R 5′-GGACTACHVGGG TWTCTAAT-3′, where barcode is an eight-base sequence unique to each sample. PCR reactions were performed in triplicate 20 μL mixture containing 4 μL of 5× FastPfu buffer, 2 μL of 2.5 mM dNTPs, 0.8 μL of each primer (5 μM), 0.4 μL of FastPfu polymerase and 10 ng of template DNA. The resulted PCR products were extracted from a 2% agarose gel and further purified using the AxyPrep DNA Gel Extraction Kit (Axygen Biosciences, Union City, CA, USA) and quantified using QuantiFluor-ST (Promega, Madison, WI, USA) according to the manufacturer’s protocol. Sequenced raw reads were deposited into the NCBI sequence read archive database (Accession Number: SRP151958). Illumina MiSeq sequencing and processing of sequencing data were conducted as previously described [25].

## RESULTS

### Hydrolytic activities of XMosidases *in vitro*

When feed ingredients were hydrolyzed by NSPase (xylanase and β-mannanase) with the addition of either XMosidases, the release of non-reducing sugar from the hydrolysis was increased. The hydrolysis rate was dependent on the dose of XMosidases. A slight synergy was observed for β-xylosidase (5 U) and β-mannosidase (10 U) (Table 2).

### *In vitro* simulation of gastric and small intestinal digestion by XMosidases

Analysis of β-xylosidase and β-mannosidase hydrolysis of feed ingredients in an *in vitro* simulation system increased the digestion of dry matter (DM) (Table 3). The combination of β-xylosidase (5 U) and β-mannosidase (10 U) to NSPase achieved 67.89%±0.22% DM digestibility and 63.12%±0.21% digestible energy.

### Additional XMosidases improved growth performance and apparent total tract digestibility in weanling pigs

We next examined the effects of β-xylosidase and β-mannosidase on growth performance and ATTD in weanling pigs (Table 4). In the entire phase (d 0 to 28 of the experiment) pigs fed the control diet were compared with both pigs fed the CE and the combination of β-xylosidase, β-mannosidase. The CE increased final BWs (p = 0.04) with the CE-XMosidases group showing the greatest increase. Similar results for ADG were obtained (p = 0.048). Daily feed intake was not significantly different (p>0.05) between groups. Feed conversion rate (F:G) of the CE-XMosidases group was lower than that of the control and CE groups (p = 0.049). The ATTD of crude protein (CP) and DM were increased by addition of β-xylosidase and β-mannosidase to CE supplemented diets.

### Diversity of bacterial community in pig digesta of ileum, cecum, and colon

**Figure 1:**
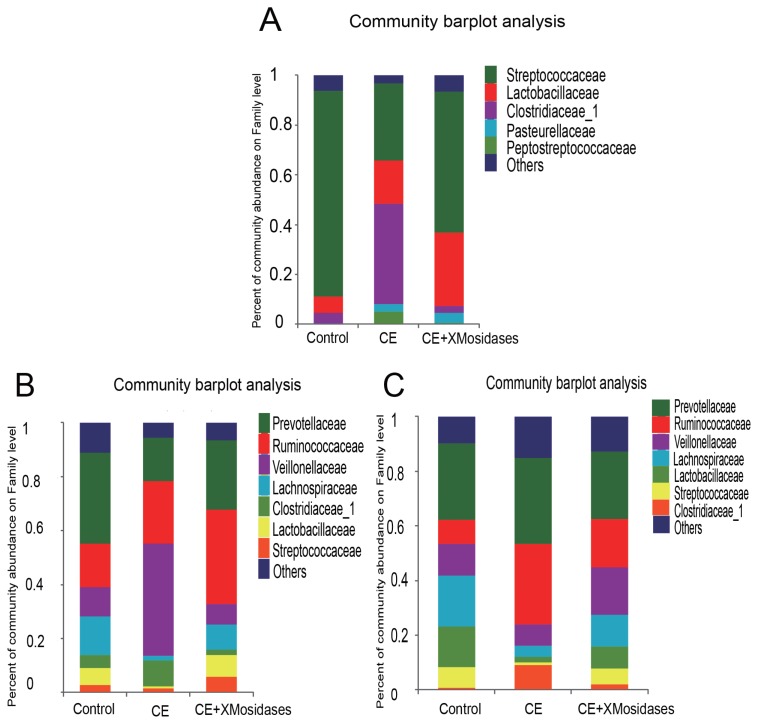
Effects of XMosidases supplementation on gut bacterial community at the family level of weanling pigs. Relative read abundance of different bacterial families in the digesta of ileum (A), cecum (B), and colon (C) in the treatments (Control, CE, CE+XMosidases). Families with proportion less than 1% were not listed. Control was the unsupplemented group. CE was the group receiving a diet supplemented with a multi–enzyme complex containing xylanase, β-mannanase, protease and amylase. CE+XMosidases, CE was the group receiving a diet supplemented with a multi-enzyme complex; and XMosidases (β-xylosidase and β-mannosidase).

**Figure 2:**
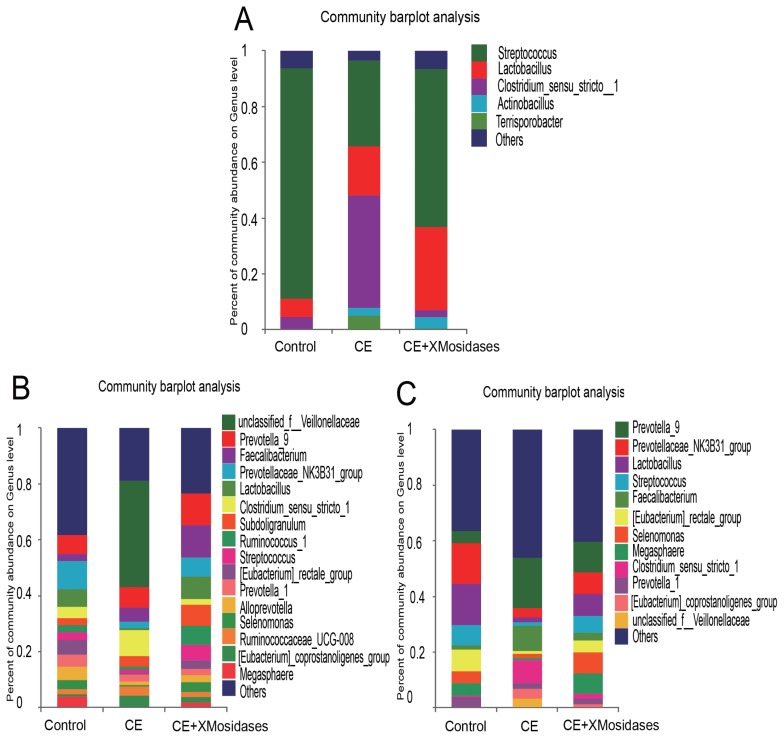
Effects of XMosidases supplementation on gut bacterial community at the genus level of weanling pigs. Relative read abundance of different bacterial genera in the digesta of ileum (A), cecum (B), and colon (C) in the treatments (Control, CE, CE+XMosidases). Genera with proportion less than 1% were not listed. Right panels demonstrated the alteration proportion of bacteria. Control was the unsupplemented group. CE was the supplemented group with multiple enzymes contained xylanase, β-mannanase, protease, and amylase in diets. CE+XMosidases, CE was the supplemented group with multiple enzymes; and XMosidases (β-xylosidase and β-mannosidase).

We further examined changes in gut bacterial composition due to dietary CE and XMosidases supplementation via sequencing of the digesta of ileum, cecum, and colon (Figures 1, 2). The profiles of bacterial composition of the CE+ XMosidases group were different from the CE group but similar to the control group in the three intestinal regions.

In the ileum, operational taxonomic unit (OTU) numbers (sobs) were 170, 97, and 164 for control, CE and CE+ XMosidases groups respectively (Table 5). At the class level, the combined relative abundance of Bacilli and Clostridia was over 90% in all groups. Clostridia represented 45.9% relative abundance in the CE group, while the proportion of Clostridia in Control group was 6.7% and in CE+XMosidases group was 4.5%. At the genus level, *Lactobacillus*, *Streptococcus*, and *Clostridium_sensu_stricto*, accounted for 90% of the total bacteria in all groups. CE (17.5%) and CE+XMosidases (29.6%) had the percentage of *Lactobacillus* compared with Control (6.6%). The abundance profile of Bacilli-Streptococcaceae-*Streptococcus* in CE group (40.4%) was also distinguished from Control (82.8%) and CE+XMosidases (56.8%).

In the cecum, the numbers of individual OTU identified in the three groups were 445, 377, and 378 respectively, but tended to be similar among groups. At the family level, the profile of CE digesta bacteria showed high proportions of Veillonellaceae (41.9%) and Lachnospiraceae (1.8%), relative to the control group where the proportions of Veillonellaceae and Lachnospiraceae were 10% and 12.6% respectively. *Lactobacillus* proportion in CE was around one tenth of the *Lactobacilli* in the other two groups.

The colon bacterial composition profile in Control and XMosidases groups were similar to each other, and were characterized by a larger proportion of Ruminococcaceae and a smaller proportion of Lachnospiraceae. This was consistent with the results in the cecum. The proportions of the colonic digesta of the CE group were Ruminococcaceae (29.5%), Veillonelaceae (7.9%), and Lachnospiraceae (4.1%). CE+ XMosidases supplementation resulted in larger proportions of *Lactobacillus*, *Selenomonase*, *Megasphaera*, *Streptococcus* and smaller proportions of *Prevotella_9, Faecalibacterium*, *Clostridium_sensu_stricto_1*. This profile was similar to that in cecal digesta.

### Volatile fatty acid concentrations in cecum and colon digesta

The lactate level in the cecum digesta of the CE supplemented group was significantly lower than that of the control and CE+ XMosidases groups (p<0.05), indicating a decreased growth of lactate producing microbes enriched in the cecum. The VFA levels in colonic digesta of three groups showed no significant differences (Table 6).

## DISCUSSION

As cereal feedstuffs, corn and SBM are abundantly utilized in feed manufacture in China. They are rich in protein and starch but also contain components resistant to mammalian digestive enzymes. Corn starch contains many complexed NSPs which link the xylose backbone and arabinose-galactose branches via glucuronic acid and ferulic acid. This structure protects corn starch from hydrolytic enzymes. Corn prolamins resemble wheat gluten immunodominant peptides which are also resistant to gastrointestinal proteolysis [26]. In this study, supplementation of a mixture of complex enzymes containing proteases, amylases, xylanases, and glucanases to a corn and SBM based diet was expected to augment the digestion rate of nutrients. The results support the hypothesis since weanling pigs fed the diets containing CE mixture and the addition of XMosidases (β-xylanosidase and β-mannanosidase) showed further enhancement of growth performance. Both CP (p = 0.01) and DM (p = 0.02) were increased over the CE group.

The mechanisms of action of CE have been well studied in relation to digestion and absorption of nutrients in small intestine. Because of the digestion of protein and starch by exogenous enzymes, these nutrients are better hydrolyzed into smaller peptides and oligosaccharides, which are more easily absorbed by foregut enterocytes. These contribute to the improvement of feed efficiency and growth performance in pigs with dietary supplementation of exogenous enzymes. The hindgut encompasses a huge microbial ecosystem, up to 10^11–12^ bacteria for every gram of gut content. In fact, the host is in competition with resident microbiota for nutrients. An inverse relationship exists between animal growth performance and intestinal microbial density [27]. In addition to the effect on improvement of feed digestion, CE supplementation depolymerizes insoluble fiber to provide more absorptive soluble oligosaccharides that increased absorption by proximal intestines and caused reduction of substrates for the microbes residential in distal intestine. In this study, CE supplementation reduced OTUs numbers in ileal digesta which is an indication of reduced bacterial diversity. The combination of XMosidases and CE likely increased oligomers in the small intestine which led to increased OTUs indicating more bacteria utilized the oligomers in the small intestine.

In response to the altered substrates by CE supplementation, the compositions of some genera were changed. *Clostridium_sensu_stricto_1* was enriched in ileal, cecal and colonic digesta. *Clostridium_sensu_stricto_1* is anaerobic commensal bacteria primarily producing butyric acids. *Clostridium* species are potential pathogenic bacteria in causing intestinal disorders [28]. Similarly, Veillonellaceae are gram positive anerobic commensal bacteria which can use lactic acids and produce acetate and propionate. While the Veillonellaceae family is considered to be a pro-inflammatory family of bacteria reported increased in inflammatory bowel diseases, irritable bowel syndrome and cirrhosis patients [29–31]. The proportion of some beneficial bacteria was diminished by CE supplementation. Lactobacillus is a genus of Gram positive anaerobic bacteria producing lactic acid [32]. *Slenomonas* ferments carbohydrates into acetate and lactate [33]. *Megasphaera*, butyrate producing bacteria, is reported to be beneficial to intestinal health in pigs [34]. We assayed VFA contents in cecum and colon. Difference was only observed in lactate among groups. The alteration of bacterial profile reflects the changing of both digestable substrates of diets and its interacted bacterial growth and metabolites. This is a complexity to judge the net benefit of the supplemented enzymes and the major microbial species involved.

Reduced hindgut fermentation substrates, lower digesta viscosity, and more rapid chime emptying rate results in reduced hindgut fermentation [25,35]. XMosidases (xylosidase and mannosidase) were able to hydrolyze corn and SBM *in vitro* producing xylose, mannose or their oligosaccharides. Xylosidase acts on xylose oligomers to release xylose residues. Mannosidases addition to the catalysis of mannanase release shorter manno-oligomers from longer-chains of mannan oligomers. The products of mannan oligomers are mainly mannobiose and mannotriol [36]. It was proposed that XMosidases produced more oligosaccharides that acted as prebiotics to favor the growth of beneficial bacteria and diminish the growth of unbeneficial bacteria. Oligosaccharides released from corn and SBM have been reported to inhibit pathogenic challenge and increase lactobacillus growth, such as AX oligosaccharides [37], soybean oligosaccharides (stachyose and raffinose) [38], manno-oligosaccharides [39].

Weaning piglets are susceptible to pathogenic antigens. A well-balanced bacterial composition in the gut is of importance for piglet health through weaning period. *Escherichia coli* [40], Clostridiaceae [41], Veillonellaceae [42], under some circumstances, are inflammatory or pro-inflammatory families of bacteria. An increase of them was reported in inflammatory intestinal diseases. XMosidases addition to CE supplementation somehow maintained lower levels of these unbeneficial bacteria in the ileal and cecal digesta. Although the primary purpose of supplementation of XMosidases in this study was to improve the digestion of plant origin feedstuff, we observed an improvement of gut bacterial distribution, which was an important addition to the effects of XMosidases in improving the growth performance in weanling piglets.

## CONCLUSION

In addition to a multiple enzyme supplementation (xylanase, β-mannanase, protease, and amylase), XMosidases (β-xylosidase and β-mannosidase) can further improve the growth performance and nutrient digestion of young pigs. XMosidases also altered the gut bacterial diversity and composition by increasing the proportion of beneficial bacteria.

## Figures and Tables

**Table 1:** Ingredient composition and chemical analysis of diets for weanling pigs

Item	
Ingredient (% as fed)	
Corn	61.95
Soybean meal	15.0
Extruded soybean	7.0
Fish meal	4.7
Whey powder	7.8
Dicalcium phosphate	0.8
Limestone	1.0
Salt	0.3
L-lysine·HCl	0.3
Threonine	0.10
Methionine	0.05
Choline	0.20
Chromic oxide	0.30
Vitamin-mineral premix1)	0.50
Total	100.00
Chemical composition (as fed)2)	
Digestible energy (kcal/kg)	3,400
Metabolisable energy (kcal/kg)	3,322
Crude protein (%)	18.76
SID lysine (%)	1.14
SID methionine (%)	0.36
Calcium (%)	0.67
Available phosphorous (%)	0.33

SID lysine, standardized ileal digestible lysine; SID methionine, standardized ileal digestible methionine.

1) Premix provided the following per kg of complete diet: vitamin A, 12,000 IU; vitamin D_3_, 2,500 IU; vitamin E, 30 IU; vitamin K_3_, 3 mg; vitamin B_1_, 0.96 mg; vitamin B_2_, 5.2 mg; vitamin B_6_, 2 mg; vitamin B_12_, 0.012 mg; nicotinic acid, 40 mg; pantothenic acid, 15 mg; folic acid, 0.4 mg; biotin, 0.04 mg; choline chloride, 0.4 g; Fe, 90 mg; Cu, 10 mg; Zn, 80 mg; Mn, 16 mg; I, 0.24 mg; Se, 0.3 mg; NaCl, 4.4 g.

2) The digestible energy, metabolisable energy, SID lysine, SID methionine and available P mean calculated values, the rest are analyzed values.

**Table 2:** *In vitro* hydrolysis activity of additional β-xylosidase and β-mannosidase on feed ingredients (soybean and corn)

Treatments	Released non-reducing sugar (mg/g feed ingredients)
Control	214.84±5.19^a^
NSPase1)	233.39±7.77^b^
NSPase+5 U β-xylosidase	269.83±4.12^b^
NSPase+10 U β-xylosidase	281.33±9.97^c^
NSPase+5 U β-mannosidase	242.67±6.58^b^
NSPase+10 U β-mannosidase	250.36±11.51^b^
NSPase+5 U β-xylosidase +10 U β-mannosidase	306.12±5.45^c^

1) NSPase comprised 5 U of xylanase and 5 U of β-mannanase.

a–c Means within the same column that have no common letters are significantly different (p<0.05).

**Table 3:** *In vitro* simulation of gastric and small intestinal digestion by XMosidases on feed ingredients (soybean meal and corn)

Treatments	IVDMD (%)	IVDE (%)
Control	66.86±0.19^a^	62.23±0.33^a^
NSPase1)	67.21±0.13^a^	62.61±0.06^a^
NSPase+5 U β-xylosidase	67.63±0.08^ab^	62.93±0.14^ab^
NSPase+10 U β-xylosidase	67.61±0.10^ab^	62.88±0.17^ab^
NSPase+5 U β-mannosidase	67.45±0.26^ab^	62.73±0.30^ab^
NSPase+10 U β-mannosidase	67.58±0.09^ab^	62.82±0.03^ab^
NSPase+5 U β-xylosidase+10 U β-mannosidase	67.89±0.22^b^	63.12±0.21^b^

IVDMD, *in vitro* dry matter digestibility; IVDE, *in vitro* digestible energy.

1) NSPase comprised 5 U of xylanase and 5 U of β-mannanase.

a–b Means within the same column that have no common letters are significantly different (p<0.05).

**Table 4:** Effect of enzyme supplementation on growth performance, percent apparent total-tract digestibility of nutrients

Items	Control	CE1)	CE+XMosidases1)	SEM	p-value
Performance					
Initial body weights (kg)	7.87	7.89	7.87	0.43	1.00
Final body weights (kg)	17.95^a^	18.66^b^	18.95^c^	0.62	0.04
ADG (g/d)	413.5^a^	442.5^b^	463.3^c^	14.83	0.048
ADFI (g/d)	787.5	789.5	799.4	35.52	1.00
Feed conversion rate (F:G)	1.91^a^	1.79^ab^	1.73^b^	0.06	0.049
ATTD (%)					
CP	67.07^a^	71.92^ab^	75.24^b^	1.52	0.01
DM	74.99^a^	80.13^ab^	83.15^b^	0.65	0.02
Diarrhea rate (%)	0.57	0.87	0.57	0.20	0.36

CE, complex enzymes; SEM, standard error of the mean; ADG, average daily gain; ADFI, average daily feed intake; ATTD, apparent total tract digestibility; CP, crude protein; DM, dry matter.

1) CE, amylase, protease, xylanase, and mannanase; XMosidases, the combination of β-xylosidase and β-mannosidase.

a–b Means within the same column that have no common letters are significantly different (p<0.05).

**Table 5:** Alpha diversity estimators of digesta in enzyme supplemented weanling pigs

Estimators	Sobs1)	Shannon	Simpson	Ace	Chao	Coverage
Ileum						
Control	170	1.272966	0.574313	291.4675	276.0714	0.998433
CE2)	97	1.958494	0.227997	167.8199	139.2727	0.999206
CE+Xmosidases2)	164	2.003657	0.30147	287.8902	290.4286	0.998381
Cecum						
Control	445	4.608488	0.017993	464.2873	465.027	0.999008
CE	377	3.171147	0.163712	411.4604	412.814	0.998525
CE+Xmosidases	378	4.089857	0.035785	405.524	421.0435	0.998924
Colon						
Control	432	4.289249	0.031887	450.1514	453.4839	0.99912
CE	431	4.286142	0.037756	455.57	454.9167	0.99894
CE+Xmosidases	428	4.346701	0.029419	443.5924	443.5	0.999155

1) Sobs are the number of observed operational taxonomic units.

2) CE, complex enzymes (amylase, protease, xylanase, and mannanase); XMosidases, the combination of β-xylosidase and β-mannosidase.

**Table 6:** Volatile fatty acid contents in cecum and colon digesta in complex enzymes and XMosidases treated pigs (mg/g)

Items	Control	CE1)	CE +XMosidase1)	SEM	p-value
Cecum digesta					
Lactate	2.09^a^	0.94^b^	1.42^ab^	0.26	0.02
Acetate	3.78	4.16	4.44	0.36	0.45
Propionate	2.66	2.67	2.67	0.20	1.00
Formate	0.36	0.23	0.29	0.05	0.24
Isobutyrate	0.20	0.09	0.03	0.07	0.28
Butyrate	1.19	1.23	1.46	0.16	0.45
Isovalerate	0.50	0.46	0.31	0.08	0.25
Valerate	0.70	0.41	0.20	0.25	0.39
Total	11.47	10.18	10.78	0.79	0.53
Colon digesta					
Lactate	1.09	1.20	1.12	0.31	0.97
Acetate	3.61	6.90	4.00	1.87	0.42
Propionate	2.14	2.18	2.27	0.38	0.97
Formate	0.14	0.48	0.18	0.20	0.45
Isobutyrate	0.08	0.1	0.07	0.01	0.31
Butyrate	1.14	1.23	1.26	0.20	0.91
Isovalerate	0.41	0.46	0.73	0.21	0.53
Valerate	0.26	1.64	0.29	0.70	0.31
Total	8.87	14.19	9.91	2.89	0.41

SEM, standard error of the mean.

1) CE, complex enzymes (amylase, protease, xylanase, and mannanase); XMosidases, the combination of β-xylosidase and β-mannosidase.

a–b Means within the same column that have no common letters are significantly different (p<0.05).
